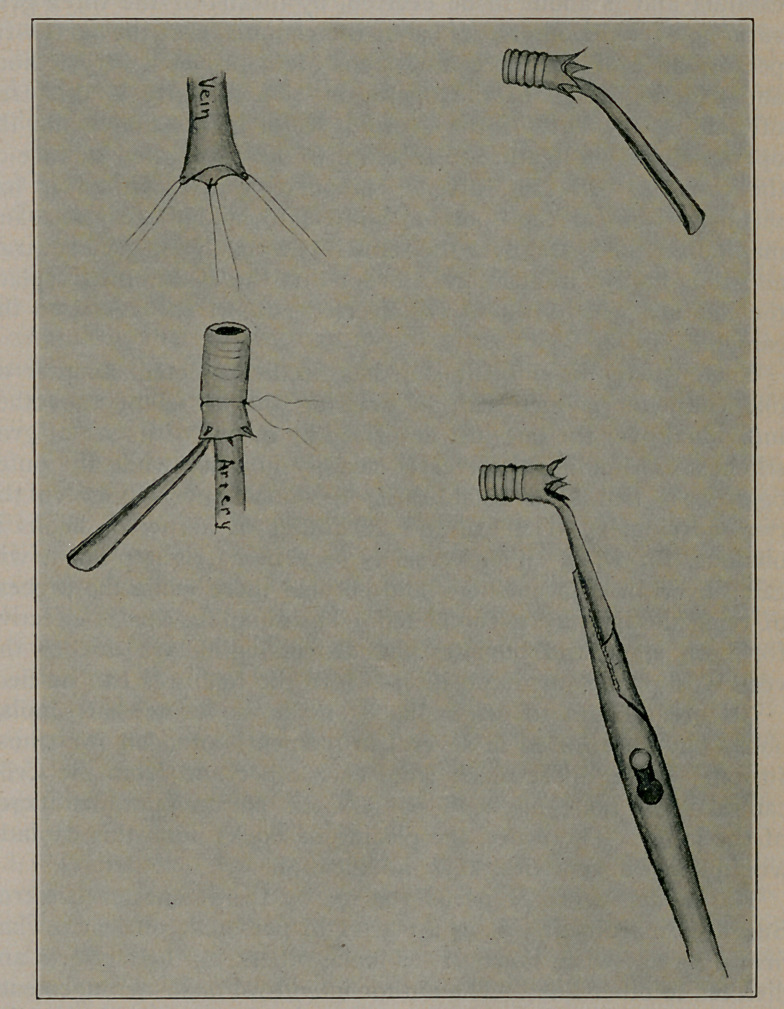# Transfusion of Blood, a Modified Instrument and a Tabulation of Indications

**Published:** 1913-12

**Authors:** Charles W. Hennington

**Affiliations:** Surgeon to the Rochester State Hospital; Surgeon to the O. P. D. Rochester General Hospital


### Transfusion of Blood, a Modified Instrument and a Tabulation of Indications

By CHARLES W. HENNINGTON, B.S., M.D.
Surgeon to the Rochester State Hospital
Surgeon to the O. P. D. Rochester General Hospital

THE proposed modification of the instrument consists in the addition of a row of hooks to the upper edge of the well known Crile Cannula, used in transfusing blood from one person to another. The illustrations accompanying this description make the matter clear at a glance. It should be observed, however, that the hooks are turned outward and that they are sharp only at the tip, and that the spaces between the hooks, as well as the lower parts of them, are well rounded off and smooth. Two styles of the idea are possible, either that the hooks be placed on the outer surface of the cannula near its upper edge, or that they be formed of the upper edge itself. This latter is the style adopted largely because of its greater simplicity of manufacture.

No lengthy explanation of the use or advantage of this modification will be attempted. Both are evident in the drawings. It is the general experience of those doing transfusion by the Crile method that after the vessel has been drawn through the cannula and is about to be everted, by means of the three grey sutures, over the outer surface of the cannula, that the successful performance of the succeeding steps often proves a very tedious and exacting stage of the operation. Particularly is this true of holding the cuff of the everted vessel in position while the operator places a ligature to fasten it to the corrugated surface of the cannula. In fact, often because of this difficulty, it has seemed wiser for the operator himself to hold the cannula and manipulate the vessel over the same by means of the grey sutures, and thus hold it in position in order that the assistant may place the ligature which fastens the everted cuff of the vessel to the cannula.

Even when this difficult step has been completed, another of like character is met with in drawing the vessel of the other individual over the one just described by means of its set of grey stitches and holding that vessel steadily in place while the outer ligature is tied, thus establishing the anastomosis between the two individuals. The use and advantage of a row of hooks is obvious, for when either vessel is once in its right place it will catch over an adjacent hook and be held there while the process of tying the ligature is completed. The simplification is effective for both steps and obviates the distressing delays due to the vessels' slipping out of position before the ligatures can be tied.

No great claim to originality is made, as somewhat similar hooks have been used in several instruments intended for transfusion. These differ much from each other and from the Crile cannula and none of them possess the paramount advantages of the latter. Therefore, the placing of hooks upon this cannula would seem a very desirable modification.

The models were prepared for me by the Rochester Electro-Surgical Instrument Co., as a matter of personal courtesy. That firm does not wish, however, to make others, as their efforts are directed to other lines. The author intends to make arrangements with another firm for the manufacture of this modified cannula.

### PART II.—THE INDICATIONS FOR TRANSFUSION.

The indications for transfusion of blood are by no means definitely established. Invariably there is much diversity of opinion in the use of any new method. This may be due on the one hand to over-enthusiasm and occasionally dishonest exploitation of the method, and on the other hand to skepticism and fear and lack of knowledge. Therefore, it seems desirable to attempt to tabulate our indications and show where we stand. Each group will be given a separate paragraph.

1. By far the most definite indication of transfusion is to supply blood in acute secondary anaemias of extreme degree. These are largely of the type of accidental hæmorrhages, including post-operative, post-partum, and the various internal hæmorrhages from special systems or organs. It is, therefore, an emergency relief when the patient is practically exsanguinated and in great jeopardy of life. Every surgical effort should be made in each case to control the local bleeding, either by ligature or otherwise. Often this can be done by a simultaneous operation with that of transfusion. It must be constantly kept in mind that if the local control is not secured, the very act of transfusion may be harmful because of raising the blood pressure and thereby causing renewed bleeding. If the bleeding has ceased of itself and an immediate operation may not seem warranted, as in a gastric ulcer, then the transfusion may be done after an interval, possibly on the following day. If the bleeding from the ulcer is extreme and repeated, then a transfusion and simultaneous operation on the ulcer is indicated.

2. The indication of next importance is that of promoting coagulation of the blood in hæmorrhage occurring in hæmophilia, jaundice, and other hæmorrhagic blood states. We have drugs and serums which should be tried first. The development of these simpler methods of promoting coagulation is very desirable. When they have failed, however, in a given case, we have in the transfusion of blood a nearly infallible method. It may be, too, that the loss of blood has become so great that transfusion is indicated on that ground alone. I think that it is granted, even by the advocates of other measures, that when these two indications are present together, namely, extreme anæmia and low coagulability, that immediate transfusion is indicated.

3. Transfusion to supplement a major surgical operation in a very anæmic patient is an indication of great promise. Without it the operator may be deterred from doing a thoroughly extensive operation at a time when it is still of value. Such a patient may be transfused just before or during such an operation. When the degree of such anæmia is known and an especially bloody operation is feared, it is wise to transfuse before or during the operation. It will be of greater value than afterwards for two reasons, namely, that the shock from loss of blood is lessened and that of the actual blood lost a certain proportion is foreign transfused blood. It is evident, therefore, that such a patient will come off the table with more of his own blood preserved than might otherwise be the case. It seems reasonable, too, to suppose that by a partial constriction of the extremities we may succeed in preserving in this way a greater proportion of the patient's own blood in that the loss in the operative field will be constituted more largely of the transfused foreign blood. This is an original suggestion of the writer. It may prove of considerable significance.

4. In the treatment of gas poisoning transfusion may be indicated on the ground of the pathology of this subject, for the hæmoglobin of the blood has formed a permanent compound with the poison and thus rendered it inactive as an oxygen carrier. The degree of poisoning could be determined by the spectroscope. Often its degree may be so evident that a transfusion should be done without delay, perhaps at the same time while we are doing artificial respiration. All that has been said applies to other similar poisons which hold the hæmoglobin inactive, such as carbon monoxide hæmoglobin, nitric oxide hæmoglobin and methæmoglobin, which latter occurs in certain coal tar product poisonings. In the milder types of these cases we give the usual treatment, but in the severe types one might bleed a little and then transfuse immediately afterward.

5. In this last group I wish to include all those ill defined and not yet well established indications for transfusion. If transfusion were a simpler operation it might be done merely to get the therapeutic effect of good, fresh blood, just as we might administer any drug or serum or organic product. The possible value of blood repeatedly given early in a case of pernicious anæmia is a question recently proposed. No one can predict as to its value in this disease, for in the past it has been given to moribund patients only and then has proved of little use. The same may apply to any aplastic anæmia. A future use of much promise is in splenic anæmia with simultaneous removal of the spleen as well. In many of these cases it is assumed that repeated transfusions would have to be done. The value of transfusion in tuberculosis is much in question at the present time, and the more prevailing opinion now seems to be against its use. Transfusion preceeded by blood-letting may be of value in certain severe toxæmias of various kinds. Thus it may be that this group will ultimately include eclampsia, pneumonia and pellagra. It is very desirable that the value of transfusion in these conditions be investigated by those who may be able to do so in a scientific way in a hospital surrounded by facilities for the accurate determination of the value of the procedure. The many failures that will occur and the many unsatisfying results may bring the method into disrepute if these trials are made in a mere random manner.

In every transfusion it is important that certain conditions should be carefully considered in each case and complied with if possible. The most important refers to the selection of the donor who should be free from transmissible disease, such as syphilis. The next also refers to the donor but with regard to his future welfare, especially with regard to the possible existence of latent disease, such as tuberculosis, which would rapidly grow worse; with regard to pregnancy if the donor is a woman; and lastly, with regard to its effects on a neurotic temperament or unstable mentality. Another matter of prime importance is the question as to possible hæmolysis or agglutination of the bloods, one to another. If this cannot be tested, close family relations, such as brother and sister, afford the greatest assurance of safety.

In transfusion we have a method compared to which there is none other. It would be unfortunate, indeed, if it fell into disrepute because of its careless routine use without thought as to indications and contra-indications and possible unfortunate results. Besides mere mastery of the technique, the operator should possess accurate knowledge of those dangers inherent in the method. Finally we must never lose sight of the great moral obligation which we are under toward the generous and trusting donor of that most precious fluid to safeguard him by all the means and knowledge at our command against a liberality which might too severely tax his vital capacity.

### Part III.—A Note on a New Method of Determining whether an Anastomosis is Actually Effective.

When the technical part of an anastomosis has been performed, everyone wonders whether the blood is actually being transfused. In the past we have watched the vein beyond the anastomosis dilate and pulsate and have hoped that these were signs that the blood was flowing. But we have placed little reliance on these signs.

Occasionally, when the opportunity offered, we have made a little arrangement to help decide this matter. This was possible only if there happened to be a branch of the main vein near the anastomosis. We have then merely clamped off that branch with a pair of artery forceps or bull-dog clamps, which we could easily remove at any time, to note whether blood would distinctly spurt from this lateral branch or merely flow as it would from any vein at all.

The new method which I am proposing occurred to me before a recent transfusion, and I feel convinced of its value. When one auscultates over the recipient's brachial vein at any point beyond the elbow where the anastomosis is usually made, a double sound is heard, which in some manner corresponds to the donor's heart beats and which can be stopped by merely pressing upon either of the vessels of the anastomosis. We have repeated this many times and invariably heard the sounds when the transfusion was effective and could stop them at will by shutting off the flow. Then, as soon as the finger was again released, the sounds reappeared. By this test we seem to be able to prove that a given anastomosis is actually effective.

---

FIBROMATOSIS OF THE STOMACH. Alexis Thomson and James Graham, *Edinburgh Med. Journ.*, July, 1913.

The absence of characteristic granulation tissue and of endarteritis and a negative Wassermann reaction exclude syphilis through the possibility of a gumma of the pylorus causing stenosis is admitted.

A specimen showing cancer and tuberculosis led to the conclusion that fibromatosis was neither directly tuberculous nor due to attenuated tubercular infection described by Poncet. Fibromatis may occur without cancer. Fibromatosis is an innocent affection of the stomach and is invariably associated with an ulcer.

When fibromatosis is associated with a deep punched-out ulcer, the surrounding mucosa may be normal. This appears to indicate that submucous fibromatosis is not the cause of the overlying ulceration.

The changes in the mucosa are primary and the submucous fibromatosis is secondary. The diffusion of the fibromatosis from the submucosa into the mucous coat, especially along the lesser curvature, and its sudden arrest at the pyloric ring, suggest that some irritant toxin is absorbed from the ulcer and in its passage along the lymphatics sets up this marked reaction.

Since ulcer precedes fibromatosis it is fairly common to find cancer as well. Attention is directed to the interesting observation that fibromatosis of the duodenum has not been recorded. The duodenum, as we know, is practically immune to cancer, in spite of the great frequency of ulceration.

In fibromatosis there is often a palpable tumor, and usually no free hydrochloric acid, so the diagnosis of cancer is confidently assumed. Owing to the difficulty of naked eye differentiation from cancer and the total unreliability of "while-you-wait histology," the authors advise resection of the affected parts.

## Figures and Tables

**Figure f1:**